# Automatic and Accurate Calculation of Rice Seed Setting Rate Based on Image Segmentation and Deep Learning

**DOI:** 10.3389/fpls.2021.770916

**Published:** 2021-12-14

**Authors:** Yixin Guo, Shuai Li, Zhanguo Zhang, Yang Li, Zhenbang Hu, Dawei Xin, Qingshan Chen, Jingguo Wang, Rongsheng Zhu

**Affiliations:** ^1^College of Engineering, Northeast Agricultural University, Harbin, China; ^2^College of Arts and Sciences, Northeast Agricultural University, Harbin, China; ^3^Agricultural College, Northeast Agricultural University, Harbin, China

**Keywords:** rice grain identification, computer vision, deep learning, rice seed setting rate, image segmentation

## Abstract

The rice seed setting rate (RSSR) is an important component in calculating rice yields and a key phenotype for its genetic analysis. Automatic calculations of RSSR through computer vision technology have great significance for rice yield predictions. The basic premise for calculating RSSR is having an accurate and high throughput identification of rice grains. In this study, we propose a method based on image segmentation and deep learning to automatically identify rice grains and calculate RSSR. By collecting information on the rice panicle, our proposed image automatic segmentation method can detect the full grain and empty grain, after which the RSSR can be calculated by our proposed rice seed setting rate optimization algorithm (RSSROA). Finally, the proposed method was used to predict the RSSR during which process, the average identification accuracy reached 99.43%. This method has therefore been proven as an effective, non-invasive method for high throughput identification and calculation of RSSR. It is also applicable to soybean yields, as well as wheat and other crops with similar characteristics.

## Introduction

Rice (Oryza sativa) is a cereal grain and the most widely consumed staple food for a large part of the world’s human population, especially in Asia (Ghadirnezhad and Fallah, 2014). The number of rice grains per panicle is a key trait that effects grain cultivation, management, and subsequent yield (Wu et al., 2019). The grains per panicle are usually divided into two categories, one is full grain and the other is empty grain. Among them, full grain is the real measure of the number of grains per panicle, and the ratio of full grain to the total number of grains per panicle is called the seed setting rate. The number of grains per panicle and the seed setting rate are considered to be the two most important traits directly reflecting rice yield (Oosterom and Hammer, 2008; Gong et al., 2018).

Generally, grain weight, grain number, panicle number, and RSSR are considered to be the main factors affecting rice yield. However, research into RSSR is improving with the advancements in science and technology. Li et al. (2013) have shown that the domestication-related POLLEN TUBE BLOCKED 1 (PTB1), a RING-type E3 ubiquitin ligase, positively regulates the rice seed setting rate by promoting pollen tube growth. Xu et al. (2017) proposed that OsCNGC13 acts as a novel maternal sporophytic factor required for stylar [*Ca*^2^]_*cyt*_ accumulation, ECM components modification, and STT cell death, and thus facilitates the penetration of the pollen tube for successful double fertilization and seed setting in rice. Xiang et al. (2019) reported on a novel rice gene, LOW SEED SETTING RATE1 (LSSR1), which regulates the seed setting rate by facilitating rice fertilization. Through these studies and their achievements, improving the RSSR has become an expected thing. However, a new issue has arisen with them, a problem posed by the automatic high-throughput calculation of the RSSR.

With developments in deep learning and plant phenotypic science, efficient and accurate research on rice through information technology (IT) has become very anticipated. Desai et al. (2019) proposed a simple pipeline which uses ground level RGB images of paddy rice to detect which regions contain flowering panicles, and then uses the flowering panicle region count to estimate the heading date of the crop. Hong Son and Thai-Nghe (2019) proposed an approach for rice quality classification. In their approach, image processing algorithms, the convolutional neural network (CNN), and machine learning methods are used to recognize and classify two different categories of rice (whole rice and broken rice), based on rice sizes according to the national standard of rice quality evaluation. Lin et al. (2018) proposed a machine vision system based on the deep convolutional neural network (DCNN) architecture to improve, compared with traditional approaches, the accuracy with which three distinct groups of rice kernel images are classified. Xu et al. (2020) proposed a simple, yet effective method termed the Multi-Scale Hybrid Window Panicle Detect (MHW-PD), which focuses on enhancing the panicle features to then detect and count the large number of small-sized rice panicles in the in-field scene. Chatnuntawech et al. (2018) developed a non-destructive rice variety classification system that benefits from the synergy between hyperspectral imaging and the deep CNN. The rice varieties are then determined from the acquired spatio-spectral data using a deep CNN. Zhou et al. (2019) developed and implemented a panicle detection and counting system based on improved region-based fully convolutional networks, and used the system to automate rice-phenotype measurements. Lu et al. (2017) proposed an innovative technique to enhance the deep learning ability of CNNs. The proposed CNN-based model can effectively classify 10 common rice diseases through image recognition technology. Chu and Yu (2020) constructed a novel end-to-end model based on deep learning fusion to accurately predict the rice yields for 81 counties in the Guangxi Zhuang Autonomous Region, China, using a combination of time-series meteorology data and area data. Xiong et al. (2017) proposed a rice panicle segmentation algorithm called Panicle-SEG, which is based on the generation of simple linear iterative clustering super pixel regions, CNN classification, and entropy rate super pixel optimization. Kundu et al. (2021) develop the “Automatic and Intelligent Data Collector and Classifier” framework by integrating IoT and deep learning. The framework automatically collects the imagery and parametric data and automatically sends the collected data to the cloud server and the Raspberry Pi. It collaborates with the Raspberry Pi to precisely predict the blast and rust diseases in pearl millet. Dhaka et al. (2021) present a survey of the existing literature in applying deep CNNs to predict plant diseases from leaf images. This manuscript presents an exemplary comparison of the pre-processing techniques, CNN models, frameworks, and optimization techniques applied to detect and classify plant diseases using leaf images as a data set.

RSSR was initially calculated manually. However, Kong and Chen (2021) proposed a method based on a mask region convolutional neural network (Mask R-CNN) for feature extraction and three- dimensional (3-D) recognition in CT images of rice panicles, and then calculated the seed setting rate through the obtained three-dimensional image. However, due to the difficulty and high cost of CT image acquisition, this method lacks practicality.

In our research, we closely link deep learning with RSSR, making it a portable tool for the automatic and high-throughput study of RSSR. Through experimental verification, we have found that the correlation between our proposed RSSROA and the results from manual RSSR calculations is as high as 93.21%. In addition, through the verification of 10 randomly selected rice panicle images, our proposed method has been shown to be able to correctly distinguish between two kinds of rice grains. The average accuracy of the number of full grains per panicle is 97.69% and the average accuracy of the number of empty grains per panicle is 93.20%. Therefore, our proposed method can effectively detect two different grains in rice panicles and can accurately calculate RSSR. It can thus become an effective method for low-cost, high-throughput calculations of RSSR.

## Materials and Methods

**FIGURE 1:**
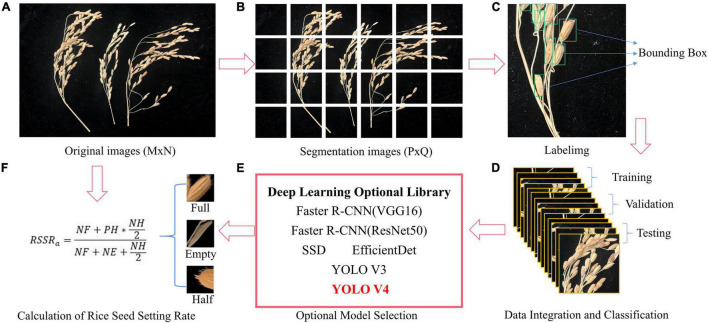
Research flow diagram. **(A)** Original images **(B)** Segmentation images **(C)** Labelimg **(D)** Data integration and classification **(E)** Optional model selection **(F)** Calculation of rice seed setting rate.

An overview of the proposed method can be seen in Figure 1. The input to our system consists of a sequence of images (across different days and times) of different rice varieties taken in a particular environment (Supplementary Table 1). The collected images were first cropped to give them the best possible resolution for the network input, and then they were input into the deep learning network we adopted for training after calibration. The training results from each network were compared, and the best network was adopted as the method to calculate the RSSR.

### Image Acquisition and Processing

Rice planting was carried out in both 2018 and 2019 at Northeast Agricultural University’s experimental practice and demonstration base in Acheng, which is located at an east longitude of 127°22′∼127°50′ and north latitude of 45°34′∼45°46′. The test soil was black soil, and there were protection and isolation rows around each 20 m^2^ plot area. The seeds were sown on April 20, 2018 (April 17 for the 2019 crop) and transplanted on May 20, 2018 (May 24 for the 2019 crop). The transplanting size was 30 cm × 10 cm and the field management was the same as for the production field (Zhao et al., 2020).

**FIGURE 2:**
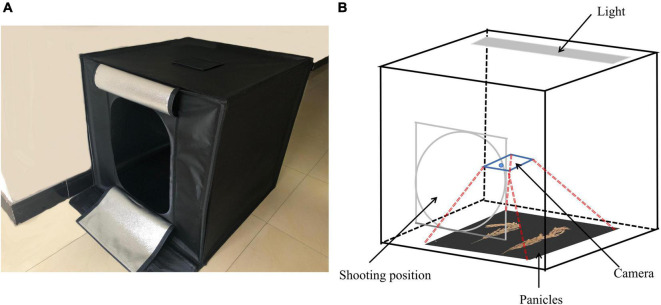
Rice panicle image collection cubed darkroom. **(A)** Real map and **(B)** structural diagram.

In order to improve the generalization ability of the experiment and reduce the time required for the artificial labeling of rice grains, 56 varieties of rice were randomly selected from the experimental field and the rice panicle information was collected using a smartphone iPhone X. The image collection environment consisted in a cubed darkroom with a length, width, and height all measuring 80 cm. The top of the darkroom environment possessed a unique light source, while the other directions were all covered by all-black light-absorbing cloth. The shooting method was to artificially push the keys on the mobile phone from the oval entrance on the front of the cubed darkroom (a rectangle measuring 55 cm in length and 40 cm in width). The shooting equipment was kept about 30 cm from the top of the rice panicles (The shooting equipment is not fixed, it only needs to be maintained manually). The image collection cubed darkroom for the rice panicles is shown in Figure 2.

A total of 263 rice panicles and 298 images were obtained. Each panicle of rice is shot in both natural and artificially shaped states. Each image contains a different panicle of rice, at least one panicle of rice and at most four panicles of rice. The panicles of each rice variety ranged from 2 to 11. Among them, 60 images were used as the data to calculate the RSSR, while the remaining images were divided into a training verification set and a test set by a ratio of 8:2.

**FIGURE 3:**
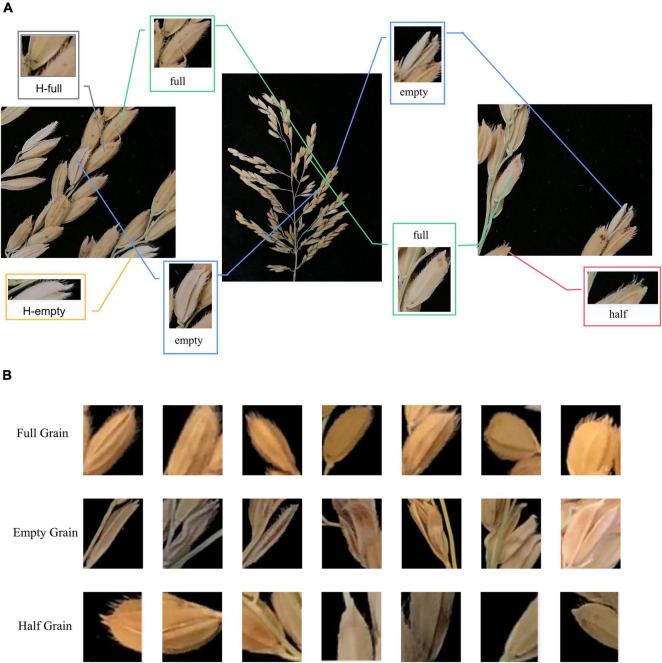
Feature image for depth learning. **(A)** Comparison of local characteristics of rice grains, **(B)** comparison of grain characteristics of different rice varieties.

We calibrated the obtained images by labeling with a target detection marking tool, and then used these images for training and prediction purposes. Figure 3A shows the calibration difference between different data sets, and Figure 3B shows the detailed differences between various categories in the image cutting process, where “full” represents a full rice grain, “empty” represents an empty rice grain, “half” represents a half rice grain, “H-full” and “H-empty” represent the full and empty grains detected in in the half grain count after cropping.

### Convolutional Neural Network

The CNN consists of several layers of neurons and computes a multidimensional function with several variables (Chen et al., 2014; Schmidhuber, 2015). The neurons in each layer, other than from the first layer, are connected with the neurons from the preceding layer. The first layer is called the input layer (Zhang et al., 2015; Dong et al., 2016), which is then followed by hidden layers, and the concluding layer. Each neuron connection has a weight that is adjusted during the learning process. Initially, the weights are taken at random. All neurons receive input values, which they then process and send out as output values. The input layer neurons’ input and output values are the values from the variables of the function. In the other layers meanwhile, a neuron receives at its input the weighted sum of the output values from the neurons with which the neuron in question is connected. The weights of the connections are used as the weights for the weighing process. Each neuron gives its function to an input value and these functions are called activation functions (LeCun et al., 2015; Mitra et al., 2017).

The motivation of building an Object Detection model is to provide solutions in the field of computer vision. The primary essence of object detection can be broken down into two parts: to locate objects in a scene (by drawing a bounding box around the object) and later to classify the objects (based on the classes it was trained on). There are two deep learning based approaches for object detection: one-stage methods (YOLO–You Only Look Once, SSD–Single Shot Detection) and two-stage approaches (Faster R-CNN) (Rajeshwari et al., 2019). In addition, we have added a newer one-stage object detector-EfficientDet. These will be our main research methods.

#### Faster Region Convolutional Neural Network

**FIGURE 4:**
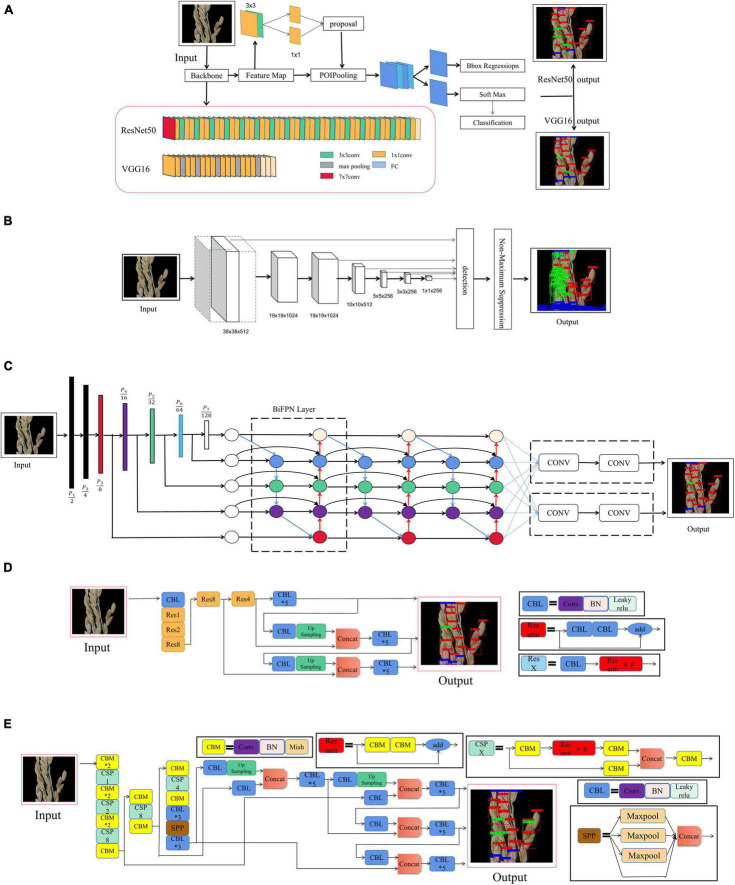
Convolutional neural network. **(A)** Faster R-CNN, **(B)** SSD, **(C)** EfficientDet, **(D)** YOLO V3, and **(E)** YOLO V4.

As a typical two-stage object detection algorithm, the faster region convolutional neural network (Faster R-CNN) has been widely applied in many fields since its proposal (Ren et al., 2016). As shown in Figure 4A, a region proposal network (RPN) is constructed to generate confident proposal for multi-classification and bounding box refinement. More precisely, RPN first generates a dense grid of anchor regions (candidate bounding boxes) with specified sizes and aspect ratios over each spatial location of the feature maps. According to intersection over union (IOU) ratio with the ground truth object bounding boxes, an anchor will be assigned with a positive or negative label on top of the feature maps, a shallow CNN is built to judge whether an anchor contains an object and predict an offset for each anchor. Then anchors with high confidence are rectified by the offset predicted in RPN. Then the corresponding features of each anchor will go through a RoI pooling layer, a convolution layer and a fully connected layer to predict a specific class as well as refined bounding boxes (Zou et al., 2020). In addition, it is worth noting that we use ResNet50 and VGG16 as the backbone networks for training.

#### Single Shot Detector

The single shot detector (SSD) (Liu et al., 2016) discretizes the bounding boxes’ output space into a set of default boxes over different aspect ratios and scales per feature mAP location. At the predicted time, the network awards scores to the situation of each object category in each default box, after which, it makes the according adjustments to the box to better match the object shape. Additionally, in order to naturally handle objects of various sizes, the network combines predictions from multiple feature mAPs with different resolutions. SSD is simple compared to methods that require object proposals, because it completely eliminates the need for proposal generations and the subsequent pixel or feature resampling stages, and encapsulates all the necessary computations in a single network. This makes SSD easily trainable and straightforward to integrate into systems requiring a detection component (see Figure 4B).

#### EfficientDet

EfficientDet proposes a weighted bi-directional feature pyramid network (BiFPN) and then uses it as the feature network. It takes level 3–7 features (P3, P4, P5, P6, P7) from the backbone network and repeats the top-down and bottom-up bi-directional feature fusion. These fused features are fed to the class and box networks to generate object class and boundary box predictions, respectively. A composite scaling extension method is also proposed, which is able to uniformly scale the resolution, depth and width of all the backbone networks, feature networks and prediction networks. The network structure of EfficientDet is shown in Figure 4C (Tan et al., 2020).

#### You Only Look Once

YOLO V3 adopts a network structure called Darknet53. It draws on the practice of residual network, and sets up fast links between some layers to form a deeper network level and multi-scale detection, which improves the detection effect of mAP and small objects (Redmon and Farhadi, 2018). Its basic network structure is shown in Figure 4D.

The real-time and high-precision target detection model, YOLO V4, allows anyone training and testing with a conventional GPU to achieve real-time, high quality and convincing object detection results. As an improved version of YOLO V3, YOLO V4 combines many of the techniques from YOLO V3. Among them, the feature extraction network, Darknet53, which was the backbone network for YOLO V3, has been changed to CSPDarknet53, the feature pyramid has become SPP and PAN, while the classification regression layer remains the same as in YOLO V3. In order to achieve better target detection accuracy without increasing inference costs, a method is used that either only changes the training strategy or only increases the training cost. This method is called the “bag of freebies.” A common method for target detection that meets the requirements of being a “free bag” in the “bag of freebies” method, is data enhancement. The purpose of data augmentation is to increase the variability of the input images, meaning that the designed object detection model will have higher robustness to images obtained in different environments. Another addition to this method, is known as the “bag of specials.” This bag consists of plugin modules and a post-processing method that can significantly improve the accuracy of object detection and only increase the inference cost by a small amount. Generally speaking, these plugin modules are used to enhance certain attributes in a model, such as enlarging the receptive field, introducing an attention mechanism, or strengthening feature integration capability. Post-processing meanwhile, consists in a method used for screening model prediction results. Its basic network structure is shown in Figure 4E (Bochkovskiy et al., 2020).

### Hardware and Software

The CNNs were trained on the rice image dataset using a hardware solution from our computer. This was a personal desktop computer with Intel core i9-9900k CPU, NVIDIA Titan XP (12G) GPU, and 64G RAM. We used the desktop to train the six networks in Python language under a Windows operating system with a Pytorch framework.

### Rice Seed Setting Rate Optimization Algorithm

Obtaining the RSSR is the ultimate goal of this research. According to the traditional RSSR calculation formula used in agriculture, the following formula was offered for adaption to our research results:


(1) $$R\,S\,S\,R_{t}=\frac{N\,F_{t}}{N\,F_{t}+N\,E_{t}}$$

We put forward a novel method to calculate the RSSR, which is to segment the original rice images to form the third category “half grain,” and calculate the RSSR by finding the correlation among them. This method is called the rice seed setting rate optimization algorithm (RSSROA), the formula is as follows:


(2) $$R\,S\,S\,R_{a}=\frac{N\,F+P\,H\times \frac{N\,H}{2}}{N\,F+N\,E+\frac{N\,H}{2}}$$

(3) $$R\,a\,t\,i\,o_{1}=\frac{N\,F}{N\,F+N\,E}$$

(4) $$R\,a\,t\,i\,o_{2}=\frac{N\,F\,H}{N\,F\,H+N\,E\,H}$$

where *RSSR*_*t*_ is a traditional measurement method used for calculating the RSSR in agronomy, *NF*_*t*_ is the number of full grains obtained by traditional methods, *NE*_*t*_ is the number of empty grains obtained by traditional methods, *RSSR*_*a*_ is the RSSR result calculated by our rice seed setting rate optimization algorithm (RSSROA), *NF*(*NUMBEROFFULLGRAIN*) is the number of full rice grains obtained by RSSROA, *NE*(*NUMBEROFEMPTYGRAIN*) is the number of empty grains obtained by RSSROA, *NH*(*NUMBEROFHALFGRAIN*) is the number of half grains obtained by RSSROA, *PH*(*PROBABILITYOFFULLHALFSEED*) is the prior probability of there being full grains of rice in the half grain count, *NFH*(*NUMBEROFFULLGRAININHALFGRAIN*) is the number of full grains in the half grain count, and *NEH*(*NUMBEROFEMPTYGRAININHALFGRAIN*) is the number of empty grains in the half grain count.

**FIGURE 5:**
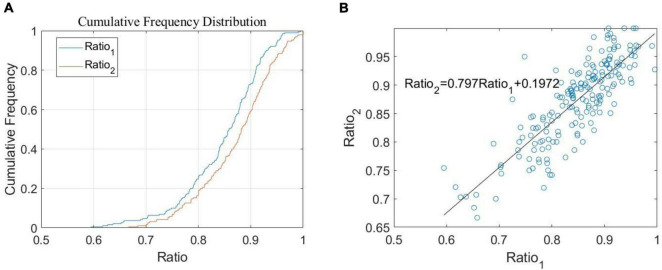
Research on the relationship of Ratio. **(A)** The proportion of cumulative frequency according to the change of ratio **(B)** relationship between *Ratio*_1_ and *Ratio*_2_.

Through our simulation study, it was found that there is a certain linear relationship between *Ratio*_1_ and *Ratio*_2_. This can be seen in Figure 5A, which shows the distribution density curves of *Ratio*_1_ and *Ratio*_2_, where both curves belong to normal distribution and have 99.89% probability of consistency by the Kolmogorov-Smirnov test (Frank, 1951). Therefore, we further explored and obtained the scatter diagram with *Ratio*_1_ as the *X*-axis and *Ratio*_2_ as the *Y*-axis, as shown in Figure 5B. Through a correlation analysis, we then obtained the correlation coefficient of 0.8327 and the linear equation of *PH* = *Ratio*_2_ = 0.797*Ratio*_1_+0.1972. The result of this current method can be used as our *PH* coefficient.

### Evaluation Standard

We evaluated the results from the different networks used on our data set. For the evaluation, a detected instance was considered a true positive if it had a Jaccard Index similarity coefficient, also known as an intersection-over-union (IOU) (He and Garcia, 2009; Csurka et al., 2013) of 0.5 or more, with a ground truth instance. The IOU is defined as the ratio of pixel number in the intersection to pixel number in the union. The instances of ground truth which did not overlap with any detected instance were considered false negatives. From these measures, the precision, recall, F1 score, AP, and mAP were calculated (Afonso et al., 2020):


(5) $$P\,r\,e\,c\,i\,s\,i\,o\,n=\frac{T\,P}{T\,P+F\,P}$$

(6) $$R\,e\,c\,a\,l\,l=\frac{T\,P}{T\,P+F\,N}$$

(7) $$F\,1=\frac{2\,P\,r\,e\,c\,i\,s\,i\,o\,n\times R\,e\,c\,a\,l\,l}{P\,r\,e\,c\,i\,s\,i\,o\,n+R\,e\,c\,a\,l\,l}$$

(8) $$A\,P=\sum_{k=1}^{N}P\,r\,e\,c\,i\,s\,i\,o\,n\,\left(k\right)\,△\,R\,e\,c\,a\,l\,l\,\left(k\right)$$

(9) $$m\,A\,P=\frac{\sum_{i}^{M}A\,P_{i}}{M}$$

where *TP* = the number of true positives, *FP* = the number of false positives, and *FN* = the number of false negatives. Where *N* is the total number of images in the test dataset, *M* is the number of classes, *Precision*(*k*) is the precision value at *k* images, and △*Recall*(*k*) is the recall change between the *k* and *k-1* images.

In addition, the mean absolute error (*MAE*), the mean squared error (*MSE*), the root mean squared error (*RMSE*), and the correlation coefficient (*R*), were used as the evaluation metrics to assess the counting performance. They take the forms:


(10) $$M\,A\,E=\frac{1}{N}\,\sum_{1}^{N}\left|t_{i}-c_{i}\right|$$

(11) $$M\,S\,E=\frac{1}{N}\,\sum_{1}^{N}{\left(t_{i}-c_{i}\right)}^{2}$$

(12) $$R\,M\,S\,E=\sqrt{\frac{1}{N}\,\sum_{1}^{N}{\left(t_{i}-c_{i}\right)}^{2}}$$

(13) $$R=\sqrt{1-\frac{\sum_{i=1}^{N}{\left(t_{i}-c_{i}\right)}^{2}}{\sum_{i=1}^{N}{\left(t_{i}-\bar{t}\right)}^{2}}}$$

where *N* denotes the number of test images, *t*_*i*_ is the ground truth count for the *i-th* image, *c_i_* is the inferred count for the *i-th* image, and $\bar{t}$ is the arithmetic mean of *t*_*i*_.

## Results

### Rice Grain Detection

**FIGURE 6:**
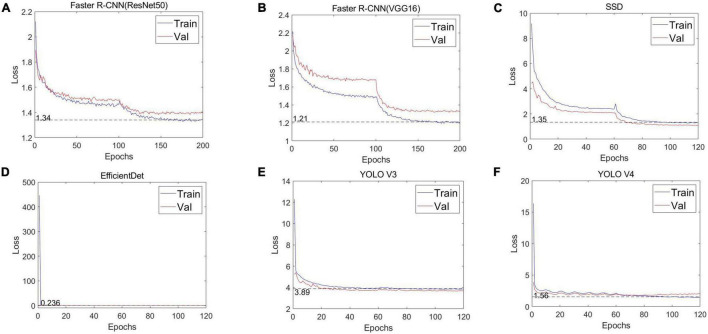
Loss curves of the different CNNs. **(A)** Faster R-CNN (ResNet50), **(B)** Faster R-CNN (VGG16), **(C)** SSD, **(D)** EfficientDet, **(E)** YOLO V3, and **(F)** YOLO V4.

First, we evaluated the convergence between the YOLO series model (YOLO V3, YOLO V4) and its four alternatives [Faster R-CNN (ResNet50), Faster R-CNN (VGG16), SSD, and EfficientDet], as well as the number of iterations. The loss curves of the training and verification processes from the adopted six deep neural networks are shown in Figure 6. For the full six networks, the uniform batch size is 4 and the learning rate starts from 0.0001. In terms of iterations, 200 are used for Faster R-CNN (ResNet50) and Faster R-CNN (VGG16), while SSD, EfficientDet, YOLO V3 and YOLO V4 use 120. It can be seen that at the beginning of the training phase, the training loss drops sharply, and then after a certain number of iterations, the loss value slowly converges around an accurate value.

Liu et al. (2021) proposes a self-attention negative feed-back network (SRAFBN) for realizing the real-time image super-resolution (SR). The network model constrains the image mapping space and selects the key information of the image through the self-attention negative feedback model, so that higher quality images can be generated to meet human visual perception. There are good processing methods for the mapping from low resolution image to high resolution image, but there is still a lack of processing method from high resolution to low resolution. Therefore, we propose the following idea: We cut the 190 images into 4,560 images, re-tagged them, and added the “half” category. Among these newly cut images, 2,705 were marked as foreground images and 1,855 were not marked as background images. We input the 2,705 foreground images into the six networks that we proposed as a data set, and obtained the precision-recall curve (Supplementary Figure 1). This greatly improved the recognition effect of all the networks (Supplementary Table 2). Among them, the mAP of the proposed YOLO V4 model in the training set reached 90.13%, which is the most effective.

The features of the full grains are that they are full and the middle of the grain presents a raised state (We believe that partially filled grains caused by abiotic stress are also full grains), empty grains meanwhile, are flat and the whole grain presents a plane effect. The three-dimensional sense in an empty grain is weaker than in a full grain, and part of the empty grain is reflected by cracks and openings in its center. The fact that these differences are small results in a poor detection effect by the alternative models we proposed. The proposed YOLO V4 model uses a Mosaic data enhancing method to reduce training costs and CSPDarknet53 to reduce the number of parameters and FLOPS of the model, which not only ensures the speed and accuracy of reasoning, but also reduces the model size. At the same time, DropBlock regularization and class label smoothing are employed to avoid any overfitting due to small differences. Thus, this means that our proposed YOLO V4 model performs much better than the other alternative models.

**FIGURE 7:**
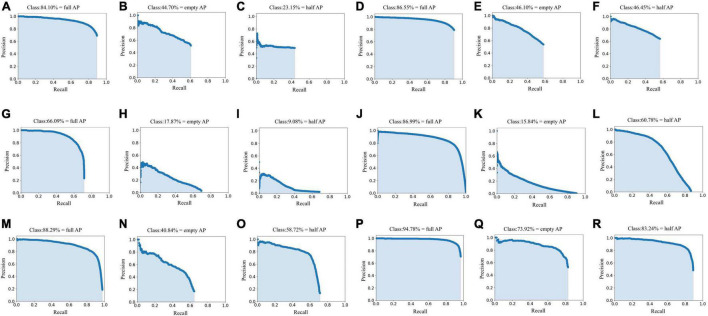
Precision-recall curves of the different convolutional neural networks in test set. **(A–C)** Are the Faster R-CNN (ResNet50) network Precision-Recall curves, where **(A)** is the full grain precision-recall curve obtained by the Faster R-CNN (ResNet50) network, **(B)** is the empty grain precision-recall curve obtained by the Faster R-CNN (ResNet50) network, and **(C)** is the half grain precision-recall curve obtained by the Faster R-CNN (ResNet50) network. **(D–F)** Are the Faster R-CNN (VGG16) network Precision-Recall curves, where **(D)** is the full grain precision-recall curve obtained by the Faster R-CNN (VGG16) network, **(E)** is the empty grain precision-recall curve obtained by the Faster R-CNN (VGG16) network, and **(F)** is the half grain precision-recall curve obtained by the Faster R-CNN (VGG16) network. **(G–I)** Are the SSD network precision-recall curves, where **(G)** is the full grain precision-recall curve obtained by the SSD network, **(H)** is the empty grain precision-recall curve obtained by the SSD network, and **(I)** is the half grain precision-recall curve obtained by the SSD network. **(J–L)** Are the EfficientDet network precision-recall curves, where **(J)** is the full grain precision-recall curve obtained by the EfficientDet network, **(K)** is the empty grain precision-recall curve obtained by the EfficientDet network, and **(L)** is the half grain precision-recall curve obtained by the EfficientDet network. **(M–O)** Are the YOLO V3 network precision-recall curves, where **(M)** is the full grain precision-recall curve obtained by the YOLO V3 network, **(N)** is the empty grain precision-recall curve obtained by the YOLO V3 network, and **(O)** is the half grain precision-recall curve obtained by the YOLO V3 network. **(P–R)** Are the YOLO V4 network precision-recall curves, where **(P)** is the full grain precision-recall curve obtained by the YOLO V4 network, **(Q)** is the empty grain precision-recall curve obtained by the YOLO V4 network, and **(R)** is the half grain precision-recall curve obtained by the YOLO V4 network.

**FIGURE 8:**
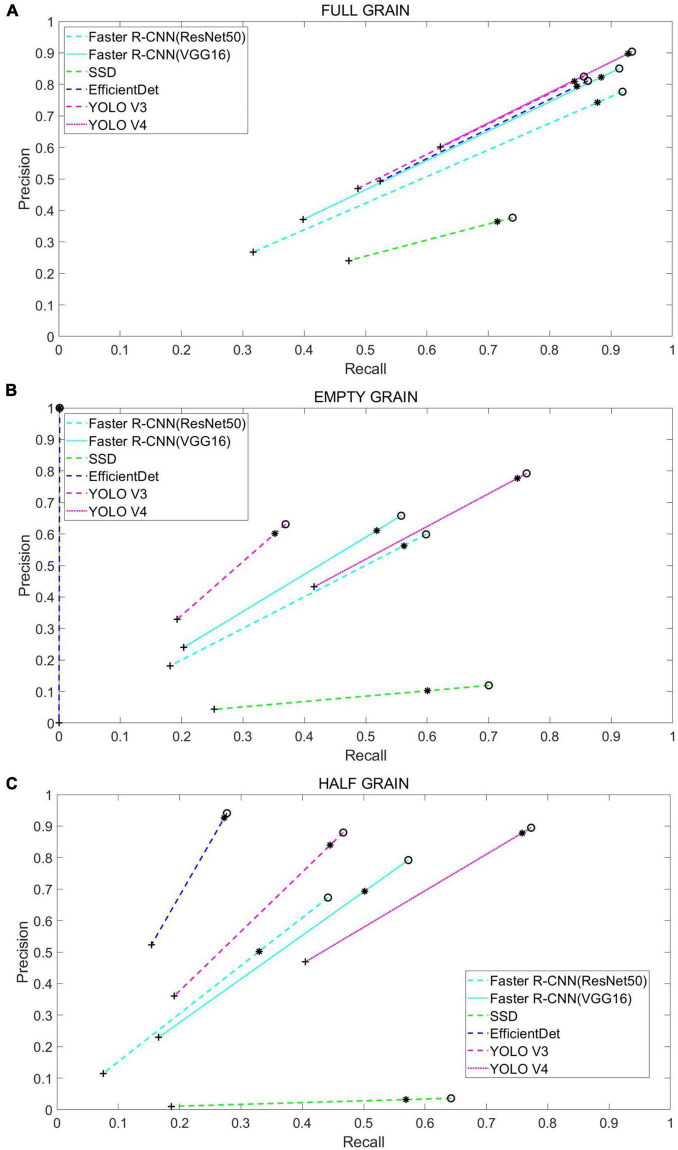
Each color corresponds to the test results from a different network model, while the symbols “°,” “*,″ and “″ correspond to a 0.25, 0.5, and 0.75 overlap IOU, respectively. The results from each method and their use of these IOU thresholds are connected by dashed lines: **(A)** Test results in full grain, **(B)** test results in empty grain, and **(C)** test results in half grain.

Following this, we tested the performance of different networks on the test set (Table 1 and Figure 7), where we plotted the precision and recall index graphs for full grain, empty grain, and half grain, with the *X*-axis corresponding to recall and the *Y*-axis corresponding to precision (Figure 8). Each color corresponds to the test results of a network structure. For each color, the symbols “°,” “*,” and “″ represent the respective overlapping IoU thresholds of 0.25, 0.50, and 0.75. Since in an ideal situation, both indicators will be close to 1, the best approach will be shown as close to the upper right corner as possible. It is clear from Figure 8 that the results from the YOLO V4 model were significantly better than those from the other networks, regardless of their category. For all methods, we noted that both accuracy and recall measures were lower when the overlap threshold was 0.75, and highest when the overlap threshold was 0.25. This means that in the case of more stringent matching criteria (higher IoU thresholds), fewer detected rice grains were matched with instances from the ground truth, which resulted in lower indices for both. The network closest to the top right was YOLO V4, with an overlap threshold of 0.25 and 0.50, respectively.

**TABLE 1:** Detection performance of different models in the test set during the clipping stage.

Network name	Category	Precision	Recall	F1	AP	mAP
Faster R-CNN (ResNet50)	Full grain	74.24%	87.80%	0.80	84.10%	50.65%
	Empty grain	56.28%	56.21%	0.56	44.70%	
	Half grain	50.20%	32.95%	0.40	23.15%	
Faster R-CNN (VGG16)	Full grain	82.32%	88.43%	0.85	86.55%	59.70%
	Empty grain	61.07%	51.77%	0.56	46.10%	
	Half grain	69.35%	50.16%	0.58	46.45%	
SSD	Full grain	36.43%	71.47%	0.48	66.09%	31.01%
	Empty grain	10.24%	60.05%	0.18	17.87%	
	Half grain	3.18%	56.91%	0.06	9.08%	
EfficientDet	Full grain	79.43%	84.45%	0.82	86.99%	54.54%
	Empty grain	100.00%	0.02%	0.00	15.84%	
	Half grain	92.54%	27.26%	0.42	60.78%	
YOLO V3	Full grain	81.00%	84.07%	0.83	88.29%	62.62%
	Empty grain	60.12%	35.19%	0.44	40.84%	
	Half grain	83.94%	44.54%	0.58	58.72%	
YOLO V4	Full grain	89.79%	92.79%	0.91	94.78%	83.98%
	Empty grain	77.66%	74.68%	0.76	73.92%	
	Half grain	87.79%	75.83%	0.81	83.24%	

### Calculation of Rice Seed Setting Rate

Through an analysis and comparison, YOLO V4 was finally selected as the main network to be used for RSSR predictions, due to its good partitioning effect on the rice grains. For the calculation of RSSR, the rice images were first input for automatic cropping, with the number of full grain, empty grain, and half grain in each cropped image predicted by the YOLO V4 network. Following this, all sub-images belonging to an image were automatically synthesized, and the RSSR was calculated according to the algorithm we provided.

**FIGURE 9:**
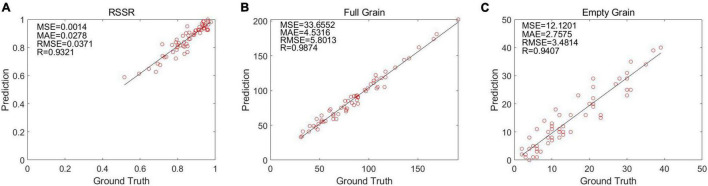
The results calculated by the algorithm are in the form of a linear regression: **(A)** Linear regression of full grains in the optimization algorithm, **(B)** linear regression of empty grains in the optimization algorithm, and **(C)** linear regression of half grains in the optimization algorithm.

The linear regression between the manual calculation result and the optimization algorithm’s calculation result of 60 rice images is shown through (Figures 9A–C). It can be observed that YOLO V4 is the most efficient at identifying rice grains, and that its correlation coefficient *R* surpasses 90%.

Table 2 is a comparison of the results from the proposed method and those that were obtained manually. From Table 2, it can be seen that the proposed method’s average accuracy for calculating the full grain number per panicle was 97.69%, for the empty grain number per panicle it was 93.20%, and for the RSSR it was 99.43%. This indicates that the proposed method offers high accuracy and stability. The deviations in a few cases can be attributed to identification errors for some small empty grains and half grains during the YOLO V4 model’s testing process. The characteristics of some empty grains are not obvious, appearing highly similar to the full grains. Some half grains have a relatively complete shape, which is similar to the shape of full grains with their shielding, resulting in recognition difficulties.

**TABLE 2:** Comparison of the proposed method’s results and those obtained manually.

Sample label	1	2	3	4	5	6	7	8	9	10
No. of full grains per panicle determined manually	64	88	117	83	97	141	54	64	52	89
No. of full grains per panicle determined using proposed algorithm	64	86	119	82	99	146	55	66	55	91
No. of empty grains per panicle determined manually	35	39	27	21	15	9	20	5	3	12
No. of empty grains per panicle determined using proposed algorithm	34	40	27	20	16	10	20	5	2	11
RSSR determined manually, %	64.65	69.29	81.25	79.81	86.61	94.00	72.97	92.75	94.55	88.12
RSSR determined using proposed algorithm, %	64.89	68.53	81.55	80.23	86.18	93.65	73.08	92.69	95.79	88.98
Accuracy of the full grain number per panicle, %	100	97.73	98.32	98.80	97.98	96.58	98.18	96.97	94.55	97.80
Accuracy of the empty grain number per panicle, %	97.14	97.50	100	95.24	93.75	90.00	100	100	66.67	91.67
Accuracy of the seed setting rate, %	99.63	98.90	99.63	99.48	99.50	99.63	99.85	99.94	98.71	99.03

## Discussion

### Detection Effect of Different Data Sets

To better understand the performance of our proposed methods, we studied the network detection effects during different image states. First, however, it must be noted that the rice identification process is carried out using the initial image, which has 4,032 × 3,024 pixels.

Table 3 shows the detection performances of the six deep learning networks, all of which are clear as the high input images undergo the necessary resizing before going through the networks. However, in spite of the preservation of various network category characteristics, the minor differences between full and empty grains are still easily ignored. Therefore, although we adopted a variety of networks to train the data set, we were still unable to find a network with an accuracy as high as our own experimental results. Our proposed model, the YOLO V4 network, achieved the best accuracy among the six networks, with an mAP value of 17.97%, however, this is still far below our target expectations.

**TABLE 3:** Detection performance of the different models during the training data set’s untrimmed state.

Network name	Category	Precision	Recall	F1	AP	mAP
Faster R-CNN (ResNet50)	Full grain	14.43%	3.01%	0.05	0.55%	0.30%
	Empty grain	6.61%	0.26%	0	0.05%	
Faster R-CNN (VGG16)	Full grain	12.47%	2.40%	0.04	0.37%	0.21%
	Empty grain	7.63%	0.22%	0	0.04%	
SSD	Full grain	9.37%	9.95%	0.1	1.11%	0.67%
	Empty grain	2.14%	0.14%	0	0.22%	
EfficientDet	Full grain	0.01%	0.01%	0	0.26%	0.14%
	Empty grain	0.01%	0.01%	0	0.01%	
YOLO V3	Full grain	45.53%	45.77%	0.46	29.82%	16.65%
	Empty grain	37.21%	4.39%	0.08	3.48%	
YOLO V4	Full grain	49.54%	40.30%	0.44	24.51%	17.97%
	Empty grain	43.69%	17.60%	0.25	11.43%	

Table 4 shows the detection effect under precise division. 4,560 images were obtained by cropping 190 images, whereupon these were used as the data set. The cropping principle is that the size of the cropped images be as close as possible to the input size of each network, and that the categories of half-full grain and half-empty grain are added. H-full and H-empty represent the full and empty grains detected in in the half grain count after cropping. It can be observed that the accuracy of all the networks and the recognition accuracy of some of the categories have been improved. These results accorded with our hypothesis and proved the effectiveness of the proposed method. However, the overall performance remains unsatisfactory.

**TABLE 4:** Detection performance of various networks under precise division.

Network name	Category	Precision	Recall	F1	AP	mAP
Faster R-CNN (ResNet50)	Full grain	73.85%	86.68%	0.80	80.82%	37.04%
	Empty grain	59.84%	43.10%	0.50	36.48%	
	H-full grain	51.31%	31.87%	0.39	25.12%	
	H-empty grain	51.54%	4.35%	0.08	5.73%	
Faster R-CNN (VGG16)	Full grain	77.89%	90.01%	0.84	86.53%	43.91%
	Empty grain	59.51%	51.42%	0.55	43.66%	
	H-full grain	75.34%	30.13%	0.43	36.66%	
	H-empty grain	73.08%	3.70%	0.07	8.77%	
SSD	Full grain	70.67%	75.72%	0.73	71.24%	37.75%
	Empty grain	38.80%	50.25%	0.44	38.99%	
	H-full grain	16.15%	55.43%	0.25	28.89%	
	H-empty grain	34.02%	10.64%	0.16	11.87%	
EfficientDet	Full grain	80.89%	80.01%	0.80	86.01%	44.38%
	Empty grain	80.14%	1.80%	0.04	32.36%	
	H-full grain	83.19%	25.71%	0.39	58.46%	
	H-empty grain	0.00%	0.00%	0.00	0.69%	
YOLO V3	Full grain	82.93%	83.06%	0.83	87.72%	46.78%
	Empty grain	65.59%	27.47%	0.39	35.51%	
	H-full grain	80.04%	39.53%	0.53	56.16%	
	H-empty grain	80.00%	1.16%	0.02	7.74%	
YOLO V4	Full grain	86.87%	93.17%	0.9	94.27%	66.57%
	Empty grain	79.30%	76.37%	0.78	78.44%	
	H-full grain	86.73%	51.07%	0.64	64.38%	
	H-empty grain	79.93%	14.99%	0.25	29.19%	

### Prediction Effect of Different Convolution Neural Networks

**FIGURE 10:**
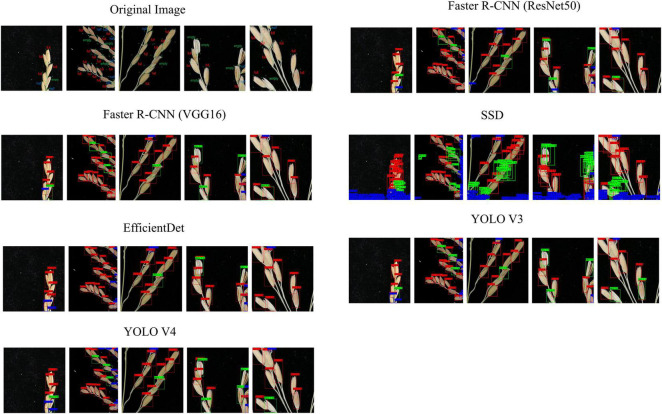
Comparison between the prediction results and the actual results from the different networks.

Figure 10 shows the predictive effects of our six network architectures: Faster R-CNN (ResNet50), Faster R-CNN (VGG16), SSD, EfficientDet, YOLO V3, and YOLO V4. Through this, it can be seen that most of the target detection methods greatly improve the detection effect once image segmentation has been completed. Faster R-CNN (ResNet50), Faster R-CNN (VGG16), EfficientDet, and YOLO V3 in particular, showed significant improvements when working with the proposed method, and performed well when detecting full grain. Almost all the full grain samples were detected, but empty and half grain samples were not detected as efficiently. YOLO V4 on the other hand, was not only the best at detecting full grains, but also at detecting the empty and half grains, as well as many categories that the other networks were unable to detect.

### Performance vs. Speed

**FIGURE 11:**
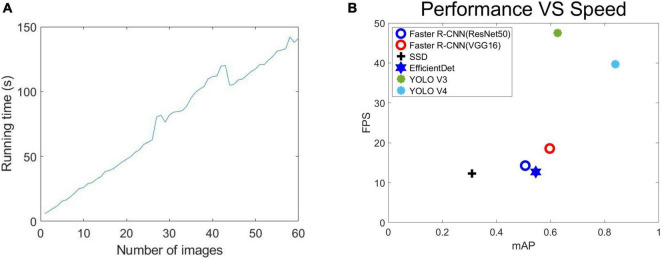
Performance: **(A)** Relationship between the number of different prediction images and prediction time, **(B)** the error in term of mAP vs. Speed (FPS) on test set.

Figure 11A shows that as the number of predicted images increased, so did the prediction time, with a roughly linear increase. We calculated that one image’s average running time is about 2.65 s, which is much less than that achieved with a manual counting time.

We also considered the reasoning speed of various networks. Figure 11B shows the error terms for mAP and speed (FPS) on the test data set. Faster R-CNN (ResNet50), Faster R-CNN (VGG16), SSD, EfficientDet, YOLO V3, YOLO V4 were all implemented using the same Pytorch framework and used the same input image size. We measured the speed of all the methods on a single Nvidia GeForce GTX TITAN XP GPU (12G) computer. According to Figure 11B, YOLO V4 is superior to the other five methods except YOLO V3 in both its speed (FPS) and mAP (the higher the better). YOLO V4 is significantly better than YOLO V3 in mAP, but the detection speed (FPS) is slightly inferior. Considering the overall situation, we think that the importance of mAP is higher than the detection speed (FPS). Therefore, we think that the performance of YOLO V4 is stronger. Faster R-CNN (ResNet50), Faster R-CNN (VGG16), and EfficientDet meanwhile, show less of a difference in their performance and speed. The SSD’s speed was similar to Faster R-CNN (ResNet50), Faster R-CNN (VGG16), and EfficientDet, but its performance was far below that of the other networks, with a poor detection of small features being the main issue.

### Error Analysis

Through the identification of the grains of 60 rice images, we detected that the average error number of full grains was 5.78 grains, and the average error number of empty grains was 2.76 grains, and the final RSSR error was 2.84%. In addition, the results of MAE, MSE, RMSE for solid grains, shrunken grains, and seed setting rates can be obtained from Figures 9A–C, which shows that although our results have certain errors, they are acceptable.

In future work, we plan to continue improving the detection accuracy of full rice grains and empty grains, and to eliminate the impact of full half grains on RSSR as much as possible. Considering the high efficiency of the program, we will also improve the RSSR calculation speed.

## Conclusion

In this paper, a RSSR calculation method based on deep learning for high-resolution images of rice panicles is proposed for the realization of the automatic calculation of RSSR. The calculation method is composed of both deep learning and RSSROA. Deep learning is used to identify the grain category characteristics of rice, and the RSSROA is used to calculate the RSSR.

In this study, a rice panicle data set composed of 4560 cut images was established. These images were taken from multiple rice varieties which had been grown under the same environment and had been processed based on image segmentation. Through the identification and comparison of data sets, we choose YOLO V4 with the best comprehensive performance as our network for calculating RSSR. In addition, the detection accuracy for full grain, empty grain, and RSSR in 10 randomly selected rice images, were 97.69, 93.20, and 99.43%, respectively. The calculation time for the RSSR in each image was 2.65 s, which meets the needs for automatic calculation. In cooperation with rice research institutions, because this method is a non-destructive operation when collecting rice panicles information, it is more convenient for rice researchers to reserve seeds, and the simple operation method enables rice researchers to obtain RSSR information more efficiently and accurately, which will be a reliable method for further estimating rice yield.

## Data Availability Statement

The datasets presented in this study can be found in online repositories. The names of the repository/repositories and accession number(s) can be found below: https://www.kaggle.com/soberguo/riceseedsettingrate.

## Author Contributions

YG: formal analysis, investigation, methodology, visualization, and writing—original draft. SL: supervision and validation. YL, ZH, and ZZ: project administration and resources. DX: writing—review and editing and funding acquisition. QC: writing—review and editing, funding acquisition, and resources. JW: writing—review and editing and resources. RZ: designed the research the article, conceptualization, data curation, funding acquisition, resources, and writing—review and editing. All authors agreed to be accountable for all aspects of their work to ensure that the questions related to the accuracy or integrity of any part is appropriately investigated and resolved, and approved for the final version to be published.

## Conflict of Interest

The authors declare that the research was conducted in the absence of any commercial or financial relationships that could be construed as a potential conflict of interest.

## Publisher’s Note

All claims expressed in this article are solely those of the authors and do not necessarily represent those of their affiliated organizations, or those of the publisher, the editors and the reviewers. Any product that may be evaluated in this article, or claim that may be made by its manufacturer, is not guaranteed or endorsed by the publisher.

## Supplementary Material

Supplementary Figure 1 Precision-recall curves of the different convolutional neural networks in training set. **(A–C)** Are the Faster R-CNN (ResNet50) network Precision-Recall curves, where **(A)** is the full grain precision-recall curve obtained by the Faster R-CNN (ResNet50) network, **(B)** is the empty grain precision-recall curve obtained by the Faster R-CNN (ResNet50) network, and **(C)** is the half grain precision-recall curve obtained by the Faster R-CNN (ResNet50) network. **(D–F)** Are the Faster R-CNN (VGG16) network Precision-Recall curves, where **(D)** is the full grain precision-recall curve obtained by the Faster R-CNN (VGG16) network, **(E)** is the empty grain precision-recall curve obtained by the Faster R-CNN (VGG16) network, and **(F)** is the half grain precision-recall curve obtained by the Faster R-CNN (VGG16) network. **(G–I)** Are the SSD network precision-recall curves, where **(G)** is the full grain precision-recall curve obtained by the SSD network, **(H)** is the empty grain precision-recall curve obtained by the SSD network, and **(I)** is the half grain precision-recall curve obtained by the SSD network. **(J–L)** Are the EfficientDet network precision-recall curves, where **(J)** is the full grain precision-recall curve obtained by the EfficientDet network, **(K)** is the empty grain precision-recall curve obtained by the EfficientDet network, and **(L)** is the half grain precision-recall curve obtained by the EfficientDet network. **(M–O)** Are the YOLO V3 network precision-recall curves, where **(M)** is the full grain precision-recall curve obtained by the YOLO V3 network, **(N)** is the empty grain precision-recall curve obtained by the YOLO V3 network, and **(O)** is the half grain precision-recall curve obtained by the YOLO V3 network. **(P–R)** Are the YOLO V4 network precision-recall curves, where **(P)** is the full grain precision-recall curve obtained by the YOLO V4 network, **(Q)** is the empty grain precision-recall curve obtained by the YOLO V4 network, and **(R)** is the half grain precision-recall curve obtained by the YOLO V4 network.
