# Supplementary figures and images for: Automatic and Accurate Calculation of Rice Seed Setting Rate Based on Image Segmentation and Deep Learning
Source: Front Plant Sci. 2021 Dec 14;12:770916. doi: 10.3389/fpls.2021.770916 (PMC8712771; doi:10.3389/fpls.2021.770916)

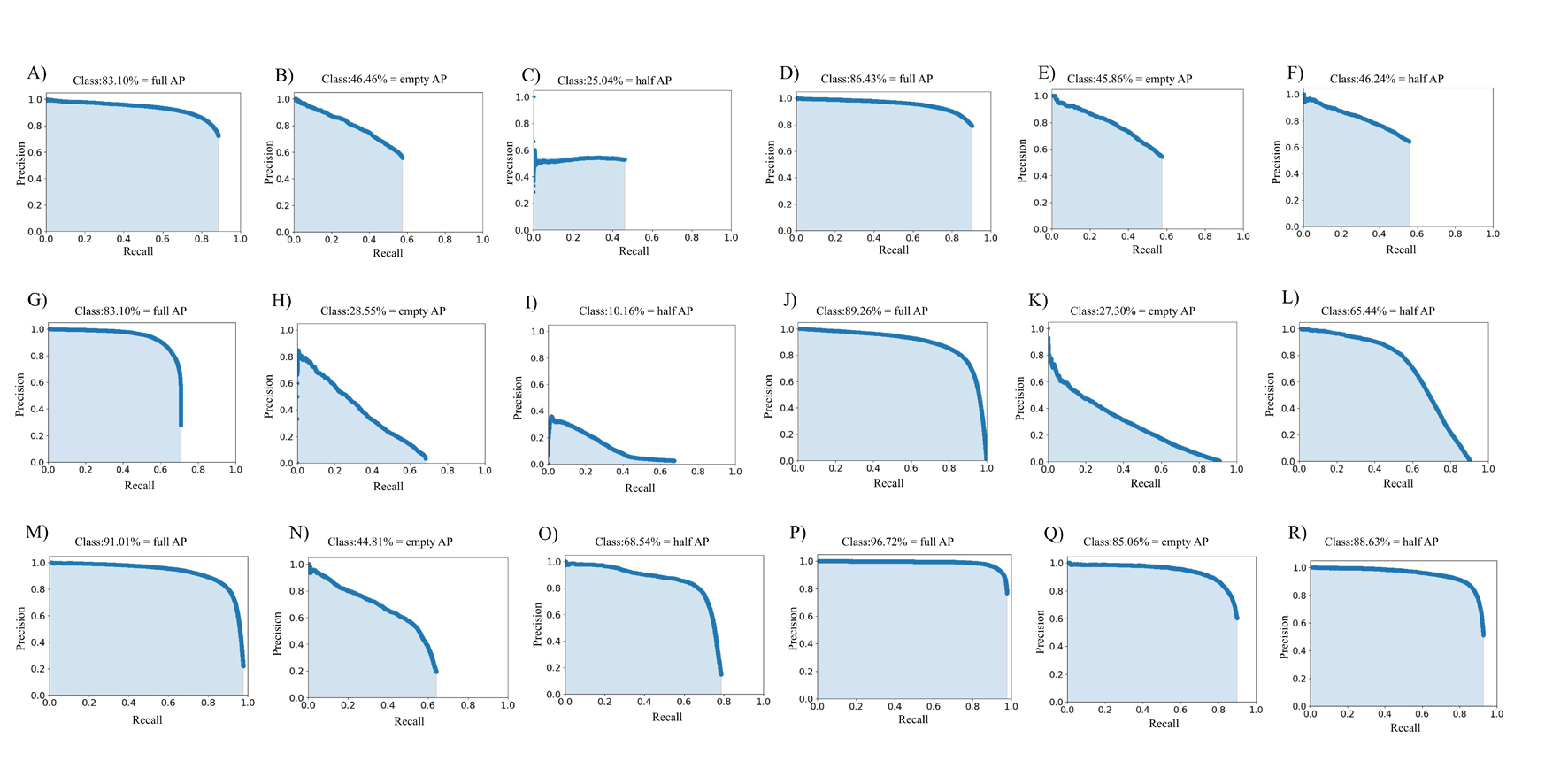

Supplement: Supplementary Figure 1 — Precision-recall curves of the different convolutional neural networks in training set. (A–C) Are the Faster R-CNN (ResNet50) network Precision-Recall curves, where (A) is the full grain precision-recall curve obtained by the Faster R-CNN (ResNet50) network, (B) is the empty grain precision-recall curve obtained by the Faster R-CNN (ResNet50) network, and (C) is the half grain precision-recall curve obtained by the Faster R-CNN (ResNet50) network. (D–F) Are the Faster R-CNN (VGG16) network Precision-Recall curves, where (D) is the full grain precision-recall curve obtained by the Faster R-CNN (VGG16) network, (E) is the empty grain precision-recall curve obtained by the Faster R-CNN (VGG16) network, and (F) is the half grain precision-recall curve obtained by the Faster R-CNN (VGG16) network. (G–I) Are the SSD network precision-recall curves, where (G) is the full grain precision-recall curve obtained by the SSD network, (H) is the empty grain precision-recall curve obtained by the SSD network, and (I) is the half grain precision-recall curve obtained by the SSD network. (J–L) Are the EfficientDet network precision-recall curves, where (J) is the full grain precision-recall curve obtained by the EfficientDet network, (K) is the empty grain precision-recall curve obtained by the EfficientDet network, and (L) is the half grain precision-recall curve obtained by the EfficientDet network. (M–O) Are the YOLO V3 network precision-recall curves, where (M) is the full grain precision-recall curve obtained by the YOLO V3 network, (N) is the empty grain precision-recall curve obtained by the YOLO V3 network, and (O) is the half grain precision-recall curve obtained by the YOLO V3 network. (P–R) Are the YOLO V4 network precision-recall curves, where (P) is the full grain precision-recall curve obtained by the YOLO V4 network, (Q) is the empty grain precision-recall curve obtained by the YOLO V4 network, and (R) is the half grain precision-recall curve obtained by the YOLO V4 n [file Image_1.TIFF]
