# Supplementary material for: Automatic and Accurate Calculation of Rice Seed Setting Rate Based on Image Segmentation and Deep Learning
Source: Front Plant Sci. 2021 Dec 14;12:770916. doi: 10.3389/fpls.2021.770916 (PMC8712771; doi:10.3389/fpls.2021.770916)
Supplement: Supplementary file 2 [file Table_1.DOCX]

Table S1. Rice data set information

| ID | Number of panicles | Number of images | ID | Number of panicles | Number of images |
| --- | --- | --- | --- | --- | --- |
| K15 | 3 | 6 | K16 | 3 | 6 |
| K17 | 3 | 6 | K18 | 3 | 6 |
| K19 | 3 | 6 | K20 | 3 | 6 |
| K21 | 3 | 6 | K22 | 3 | 6 |
| K23 | 3 | 6 | K24 | 3 | 6 |
| K25 | 3 | 6 | K26 | 3 | 6 |
| K27 | 3 | 6 | K28 | 3 | 6 |
| K29 | 3 | 6 | K30 | 3 | 6 |
| K31 | 3 | 6 | K32 | 3 | 6 |
| K61 | 11 | 8 | K62 | 9 | 6 |
| K63 | 6 | 4 | K64 | 7 | 6 |
| K65 | 7 | 4 | K66 | 5 | 4 |
| K67 | 9 | 6 | K68 | 5 | 4 |
| K71 | 4 | 5 | K72 | 6 | 6 |
| K73 | 2 | 2 | K74 | 6 | 6 |
| K75 | 4 | 4 | K82 | 7 | 8 |
| K83 | 8 | 7 | K84 | 8 | 7 |
| K85 | 7 | 6 | K86 | 4 | 4 |
| K87 | 6 | 4 | K88 | 5 | 4 |
| K89 | 5 | 5 | K90 | 2 | 2 |
| K91 | 5 | 5 | K92 | 5 | 5 |
| K93 | 4 | 4 | K94 | 3 | 3 |
| K95 | 5 | 5 | K96 | 3 | 3 |
| K97 | 4 | 5 | K98 | 4 | 6 |
| K99 | 8 | 10 | K100 | 7 | 8 |
| K101 | 3 | 2 | K102 | 6 | 4 |
| K103 | 6 | 4 | K104 | 6 | 4 |
| K105 | 9 | 6 | K106 | 4 | 4 |
