# Supplementary material for: Automatic and Accurate Calculation of Rice Seed Setting Rate Based on Image Segmentation and Deep Learning
Source: Front Plant Sci. 2021 Dec 14;12:770916. doi: 10.3389/fpls.2021.770916 (PMC8712771; doi:10.3389/fpls.2021.770916)
Supplement: Supplementary file 3 [file Table_2.docx]

Table S2. Detection performance of different models in the training set during the clipping stage

| Network name | Category | Precision | Recall | F1 | AP | mAP |
| --- | --- | --- | --- | --- | --- | --- |
| Faster R-CNN(ResNet50) | Full grain  Empty grain  Half grain | 76.43%  61.98%  53.96% | 87.44%  53.07%  36.71 % | 0.82  0.57  0.44 | 83.10%  46.46%  25.04% | 51.53% |
| Faster R-CNN(VGG16) | Full grain  Empty grain  Half grain | 82.46%  61.62%  69.60% | 88.24%  51.32%  49.79 % | 0.85  0.56  0.58 | 86.43%  45.86%  46.24% | 59.51% |
| SSD | Full grain  Empty grain  Half grain | 49.64%  14.78%  3.48% | 70.77%  59.30%  56.72% | 0.58  0.24  0.07 | 67.58%  28.55%  10.16% | 35.43% |
| EfficientDet | Full grain  Empty grain  Half grain | 81.13%  100.00%  93.32% | 85.06%  0.02%  31.19% | 0.83  0.00  0.47 | 89.26%  27.30%  65.44% | 60.67% |
| YOLO V3 | Full grain  Empty grain  Half grain | 84.77%  68.17%  87.60% | 86.39%  37.10%  51.43% | 0.86  0.48  0.65 | 91.01%  44.81%  68.54% | 68.12% |
| YOLO V4 | Full grain  Empty grain  Half grain | 93.06%  85.74%  90.47% | 94.14%  81.04%  81.14% | 0.94  0.83  0.86 | 96.72%  85.06%  88.63% | 90.13% |
